# FINANCIAL EXPLOITATION OF OLDER ADULTS: PRELIMINARY RESULTS FROM A PROSPECTIVE LONGITUDINAL STUDY

**DOI:** 10.1093/geroni/igz038.691

**Published:** 2019-11-08

**Authors:** Scott R Beach, Sara J Czaja, Richard Schulz, David Loewenstein, Peter Lichtenberg

**Affiliations:** 1 University of Pittsburgh, Pittsburgh, Pennsylvania, United States; 2 Weill Cornell Medicine, New York, New York, United States; 3 University of Miami, Miller School of Medicine, Miami, Florida, United States; 4 Wayne State University, Detroit, Michigan, United States

## Abstract

This paper presents study design and preliminary results from a new study funded by the National Institute on Aging that is examining financial exploitation (FE) among 720 White, African-American and Hispanic adults age 60+ (240 per group; 120 age 60-79; 120 age 80+). A conceptual model linking socio-demographics, physical health, social support / integration, cognitive function, financial skills / supports, and psychosocial factors to FE is being evaluated. Three assessments (baseline, 12; 24 mos.) include: a detailed cognitive battery, web-based banking simulation tasks, scam scenarios, and a standardized battery of self-report measures assessing socio-demographic and psychosocial variables. Preliminary baseline results from ~200 participants show support for the proposed model. Exposure to sales, remote purchasing behavior, and telemarketer receptivity (scam exposure); and scam vulnerability as measured by credibility ratings of “legitimate” and “fake” scam scenarios are positively associated with reports of both stranger-initiated and trusted other FE. Older adults with smaller social networks and less social support were more likely to report both exposure and vulnerability to scams. Higher general cognitive abilities, financial skills, and numeracy; and better performance on online banking tasks correlate with less scam exposure and vulnerability. Preliminary analyses of psychosocial factors also show that more depressed, impulsive, and trusting older adults report more exposure and scam vulnerability. The paper will present updated analyses of ~500 baseline participants. Understanding multiple pathways to FE is important to advance theory and for the development of interventions to minimize risk.

